# Brain glucose extraction is fixed at 10% despite twofold variability in *resting* cerebral blood flow in healthy humans

**DOI:** 10.1177/0271678X261432989

**Published:** 2026-04-07

**Authors:** Jennifer S Duffy, Hannah G Caldwell, Philip N Ainslie, Travis D Gibbons

**Affiliations:** 1 School of Physical Education, Sport & Exercise Sciences, University of Otago, Dunedin, New Zealand; 2 Centre for Heart, Lung and Vascular Health, University of British Columbia—Okanagan Campus, Kelowna, BC, Canada; 3 The August Krogh Section for Human Physiology, Department of Nutrition, Exercise and Sports, University of Copenhagen, Copenhagen, Denmark; 4 Department of Biological Sciences, Northern Arizona University, Flagstaff, AZ, USA

**Keywords:** Aerobic glycolysis, arteriovenous, cerebral blood flow, cerebral metabolism, glucose extraction, oxygen extraction

## Abstract

This commentary addresses the interpretation by Drs. Nybo and Rasmussen of our recent short report examining the interindividual variability in resting human cerebral blood flow (CBF) and metabolism in 75 otherwise healthy adult volunteers. We contend that the authors have mischaracterized the primary objective of the original short report. The objective was to quantify interindividual variability in resting CBF with oxygen and glucose extraction under resting, unstimulated conditions, rather than intraindividual regulatory responses to acute interventions. We highlight that their considerations raised do not invalidate the principal conclusion that lower resting CBF is associated with reduced aerobic glycolysis, possibly due to a greater compensatory capacity of oxygen relative to glucose extraction.

*Commentary*,

In their commentary of our recent paper in the Journal,^1^ Drs. Nybo and Rasmussen propose that this original manuscript does not represent “principal” physiology. While we appreciate this interest and scientific dialog, we strongly contend that the authors have misinterpreted the premise of our original article. They conclude that the data presented in Duffy et al., “may provide valuable insight into interindividual variability in resting physiology”, which was indeed the primary objective of this short report.^1^ The title of the original manuscript clearly states that the study focus is on “resting” human physiology. Drs. Nybo and Rasmussen further argue that the findings “do not necessarily inform the regulation of intra-individual physiological responses,” for which we agree, but again is beyond the scope of the original manuscript. The analysis was not intended to address acute regulatory mechanisms *within* individuals, but rather to characterize variability *between* individuals under resting and unstimulated conditions. Therefore, we do not find it relevant to the original manuscript to introduce ideas pertaining to the brain’s responsiveness to an acute intervention using a within-subject design. This research has been done extensively, as the authors point out.

Nevertheless, our proposition that oxygen extraction compensates *more* than glucose to low cerebral blood flow (CBF) also holds true with acute intervention in the data that Drs. Nybo and Rasmussen use to highlight their main point. From their previous research, they appropriately show that during acute CO_2_ manipulation (see their Figure 1), glucose extraction is altered. However, we caution the use of acute CO_2_ manipulations in this context since hypo- and hyper-capnia have direct effects on cerebral metabolic rates (CMR) of oxygen and glucose.^2^ Therefore, the use of hypocapnia to induce hypoperfusion when the primary outcome is glucose extraction fraction is fundamentally flawed.

We would also like to further emphasize that determining the relative variation of CMRO_2_ and CMRglc with CBF was the central objective of the contested article by Duffy et al. Previously, we reported that hypocapnia increases the oxygen-to-glucose index, evidence that oxygen extraction compensates for decreased flow more than glucose.^2^ Unlike the data provided in Figure 1 by Drs. Nybo and Rasmussen, this study includes more direct measures of both CBF and CMR and includes individual data from 41 subjects.^2^ As such, even the data provided by Drs. Nybo and Rasmussen cannot be used to refute the central findings of our contested short report; rather, their data further support it.

We acknowledge and appreciate the second issue raised in the commentary regarding the potential for a spurious relationship when correlating CMR with CBF. CBF is used in the derivation of cerebral metabolic rates and so a mathematical dependency certainly exists. However, any spurious association would depend on the extent to which extraction fraction fails to accommodate differences in CBF. Moreover the purpose of presenting the CMR–CBF regressions was to illustrate the compensatory capacity of glucose and oxygen extraction and compare to them in stoichiometric equivalents. This comparison shows the relationship between CBF and aerobic glycolysis (as a CBF independent metric), a key premise of the original manuscript.

To the authors point, any systematic error in CBF estimation would proportionally influence calculated cerebral metabolic rates. Nevertheless, such errors would be expected to be random, and therefore unlikely to systematically drive the observed correlations. Furthermore, since the intention was to compare CMRO_2_ relative to CMRglc, the CBF term within the calculations effectively cancels out, preserving the validity of the main comparison and conclusion.

To summarize, we agree fully with Drs. Nybo and Rasmussen that oxygen and glucose extraction change in response to acute interventions like hypoxia,^3^ isovolumic anemia,^3^ hypercapnia,^2^ hyperthermia,^4^ hyperglycemia,^5^ and even hypoglycemia (unpublished data by our group). But the primary research question was that amongst healthy humans, those with lower resting CBF are more likely to have lower aerobic glycolysis, and this is related to oxygen extraction having greater compensatory capacity compared to glucose extraction. Although we read this editorial with great enthusiasm, it does not refute anything presented in the original manuscript.
